# Potential role of circulating tumor cells and cell-free DNA as biomarkers in oral squamous cell carcinoma: A prospective single-center study

**DOI:** 10.1371/journal.pone.0309178

**Published:** 2024-12-27

**Authors:** Natsuki Eboshida, Atsuko Hamada, Mirai Higaki, Fumitaka Obayashi, Nanako Ito, Sachiko Yamasaki, Ryouji Tani, Tomoaki Shintani, Koichi Koizumi, Souichi Yanamoto

**Affiliations:** 1 Department of Oral Oncology, Graduate School of Biomedical and Health Sciences, Hiroshima University, Hiroshima, Japan; 2 Center of Oral Clinical Examination, Hiroshima University Hospital, Hiroshima, Japan; Virginia Commonwealth University, UNITED STATES OF AMERICA

## Abstract

Metastasis in patients with oral squamous cell carcinoma has been associated with a poor prognosis. However, sensitive and reliable tests for monitoring their occurrence are unavailable, with the exception of PET-CT. Circulating tumor cells and cell-free DNA have emerged as promising biomarkers for determining treatment efficacy and as prognostic predictors in solid tumors such as breast cancer and colorectal cancer. Hence, this study aimed to determine the potential role of liquid biopsy, circulating tumor cells, and cell-free DNA as biomarkers of oral squamous cell carcinoma. Thirteen patients with primary oral squamous cell carcinoma who visited our hospital between 2022 and 2023 were recruited, and plasma samples were collected from each patient preoperatively and postoperatively. We examined the relationship between the prognosis, the number of circulating tumor cells per four milliliters of peripheral blood, and the amount of cell-free DNA per milliliter of serum or the gene mutation in cell-free DNA. We observed no correlation between the number of preoperative circulating tumor cells and metastatic events. However, the number of circulating tumor cell clusters or the amount of preoperative cell-free DNA in metastatic cases was higher than that in non-metastatic cases. In oral squamous cell carcinoma, circulating tumor cell clusters or cell-free DNA levels may help inform management decisions regarding metastasis. However, further studies are required to provide a possible window for therapeutic interventions.

## Introduction

Oral cancer is the 11^th^ most common cancer worldwide [1]. Pathologically, oral squamous cell carcinoma (OSCC) is the most frequent oral cancer, constituting more than 90% of oral cancer [2]. The development of OSCC is considered to be related to genetic and environmental factors such as tobacco use, alcohol intake, and chronic inflammation [1]. The 5-year survival rate of patients with OSCC is poor, not more than 60% owing to tumor recurrence, metastasis, and drug resistance [3]. Therefore, the need for new clinical diagnostic measures for the effective management of OSCC is urgent.

Circulating tumor cells (CTCs) and cell-free DNA (cfDNA) are emerging as promising biomarkers for solid cancers such as breast cancer and colorectal cancer [4]. These biomarkers are already being utilized in the diagnosis and treatment of breast cancer and colorectal cancer. The detection of CTCs has been established as a valuable method for capturing circulating cancer cells released from tumors, thereby monitoring the progression and metastasis of cancer. On the other hand, the detection of cfDNA enables the analysis of free DNA released from cancer tissues, providing crucial insights into tumor genomic alterations and guiding personalized treatment selection for individual patients. The clinical application of these biomarkers significantly contributes to the individualization and effective monitoring of cancer treatment.

Because CTCs provide real-time information, research on detection devices and the characterization of CTCs have advanced significantly [5, 6]. There are many reports on the biology and clinical behavior of various tumors such as lung [7], breast [8], and colorectal cancer [9]. Recently, some groups have reported on CTCs in head and neck cancer [10–12]; however, they classified oral cancer as one type, despite oral cancers involving the tongue, gingival, cheek, and lip. In this study, we attempted to classify and analyze their potential in detail.

In 1948, Mandel and Métais first described the presence of circulating cell-free DNA (cfDNA) in human blood for the first time [13]. cfDNA consists of extracellular fragments of double-stranded DNA released into the blood due to cell death processes, such as apoptosis. cfDNA levels can be increased both in cancer patients and in healthy individuals for various reasons including trauma, infection, and inflammation [14]. In contrast, circulating tumor DNA (ctDNA) detection is more specifically indicative of tumors. To the best of our knowledge, no studies have reported on ctDNA detection in healthy individuals. In cancer patients, ctDNA can comprise <0.1% to 10% of cfDNA, with higher levels depending on tumor type and patient condition [15]. The fragment size of cfDNA is approximately 166 bp, while that of ctDNA is approximately 146 bp owing to the loss of the H1 linker [16]. Research on liquid biopsies has been extensively conducted over the past 10 years [17–19]; however, it has not been clinically used in oral cancer.

Inflammation-based prognostic score (IBPS) such as neutrophil-to-lymphocyte ratio (NLR), platelet-to-lymphocyte ratio (PLR), lymphocyte-to-monocyte ratio (LMR), and prognostic nutritional index (PNI) are recently reported prognostic factors in several carcinomas [20–24]. However, no comparative studies on cfDNA have yet been reported in OSCC. Hence, this study aimed to determine the potential role of liquid biopsy, CTCs, and cfDNA as biomarkers of OSCC.

## Material and methods

### Patient characteristics

Thirteen patients who visited the Department of Oral Oncology at Hiroshima University Hospital between JUN. 21, 2022 and JUL. 4, 2023 and underwent surgery for the histopathological diagnosis of OSCC were recruited for this study. Those who had undergone neoadjuvant treatment or were treated at other hospitals were excluded. The clinical characteristics of the recruited patients are summarized in Table 1. All patients provided written informed consent to participate in this study, which was approved by the ethics committee of Hiroshima University (approval number: hi-191-6). We retrospectively assessed the preoperative blood levels of albumin, platelet, C-reactive protein (CRP), neutrophil, lymphocyte, and monocyte.

**Table 1:** Profile of recruited patients.

Characteristic	N = 13	% of total
Age		
Median, years	73	-
Range	58–88	-
Sex		
Male	9	69.2
Female	4	30.8
The location of cancer		
Buccal mucosa	1	7.7
Maxillary gingiva	4	30.8
Mandibular gingiva	3	23.1
Tongue	3	23.1
Floor of mouth	2	15.4
Depth of tumor invasion		
T1	2	15.4
T2	2	15.4
T3	2	15.4
T4	7	53.8
Lymph node metastasis		
N0	8	61.5
N1	2	15.4
N2	2	15.4
N3	1	7.7
Cancer stage		
Ⅰ	2	15.4
Ⅱ	1	7.7
Ⅲ	2	15.4
Ⅳ	8	61.5
Distant metastasis after operation		
Positive	4	30.8
Negative	9	69.2
The number of CTC		
Median	4	-
Range	1–32	-
The amount of cfDNA (ng/mL)		
Median	3.4	-
Range	1.1–10.6	-

### CTC isolation from peripheral blood

CTC isolation was performed at Nihon Gene Research Laboratories (Sendai, Japan). Briefly, the peripheral blood (PB) of each patient was obtained from Nihon Gene Research Laboratories within 24–48 h of sampling and maintained at room temperature The cells were isolated using a microfluidic chip (Celsee Inc., Bio-Rad, MI, USA) [25], which contains 56,320 trapping chambers to capture tumor cells. Subsequently, fluorescent immunostaining of the isolated cells was performed using antibodies against 4’,6-diamino-2-phenylindole, cytokeratin, epithelial cell adhesion molecule, and programmed cell death-ligand 1 (PD-L1). The chip was analyzed using a semi-automated fluorescence microscope. CTCs were evaluated by positive selection for cytokeratin and EpCAM and negative selection for CD45.

### cfDNA extraction from plasma

We collected 8 ml of PB from each recruited patient in cfDNA BCT tubes to extract cfDNA (Streck, La Vista, USA). The time points were 1 week before surgery, 1 month after surgery, and before and after adjuvant treatment. The PB in the tube was centrifuged at 1,900 × g for 10 min at room temperature, and the upper layer of the supernatant containing the plasma was transferred to another tube and centrifuged at 16,000 × g for 10 min. Total cfDNA was extracted from an approximately final volume of 5 ml plasma using the MagMAX cfDNA isolation kit (Thermo Fisher Scientific, Waltham, MA, USA) according to the manufacturer’s protocol. cfDNA was eluted in 10–20 μL elution buffer, and its concentration was determined using Qubit (Thermo Fisher Scientific).

### Measurement of the fragment size of cfDNA

The cfDNA fragment size was measured using an Agilent 4200 bioanalyzer with the cfDNA kit (Agilent Technologies, Santa Clara, CA, USA). The Agilent 4200 Expert software (version 3.2) automatically calculated and determined the accurate quantitation, size, and percentage of cfDNA.

### Next generation sequence

Next generation sequencing (NGS) was performed by Macrogen, Inc. (Seoul, Korea). Briefly, genetic mutations in cfDNA were analyzed using Ion AmpliSeq™ Cancer Hotspot Panel v2 (Thermo Fisher Scientific), which those pool of primers prepared for the genomic frequently mutated “hot spot” regions in human cancer genes. The genes included in this panel were as follows: *ABL1*, *AKT1*, *ALK*, *APC*, *ATM*, *BRAF*, *CDH1*, *CDKN2A*, *CSF1R*, *CTNNB1*, *EGFR*, *ERBB2*, *ERBB4*, *EZH2*, *FBXW7*, *FGFR1*, *FGFR2*, *FGFR3*, *FLT3*, *GNA11*, *GNAS*, *GNAQ*, *HNF1A*, *HRAS*, *IDH1*, *IDH2*, *JAK2*, *JAK3*, *KDR*, *KIT*, *KRAS*, *MET*, *MLH1*, *MPL*, *NOTCH1*, *NPM1*, *NRAS*, *PDGFRA*, *PIK3CA*, *PTEN*, *PTPN11*, *RB1*, *RET*, *SMAD4*, *SMARCB1*, *SMO*, *SRC*, *STK11*, *TP53*, and *VHL*.

### Statistical analysis

All statistical analyses were performed using JMP® Pro 16.2.0 (SAS Institute Inc., Cary, NC, USA). Statistical significance was set at p < 0.05.

## Results

### Patient characteristics

The median age of the 13 recruited patients in this study was 72.7 years (range: 58–88 years). Nine patients were male (69.2%) and four were female (30.8%); two patients were in stage I (15.4%), one was in stage II (7.7%), two were in stage III (15.4%), and eight in stage IV (61.5%). The median follow-up period was 9.8 months (range: 5.4–15.9 months). During the postoperative follow-up, distant metastases were seen in four patients. The patient details are shown in Tables 1 and 2.

**Table 2:** Clinical characteristic and prognosis of recruited patients.

Patient No.	Sex	Age	Stage	Location	PFS (months)	OS (months)
		(year)				
1	F	84	ⅣA	Mandibular gingiva	1.9	22.8
2	M	74	Ⅲ	Tongue	35.5	36.8
3	F	58	Ⅰ	Buccal mucosa	10.8	36.6
4	M	64	Ⅰ	Tongue	35.1	37
5	F	77	ⅣA	Maxillary gingiva	9.3	17.1
6	M	62	ⅣA	Mandibular gingiva	32.8	35.7
7	M	76	ⅣA	Floor of mouth	33	35.7
8	M	62	Ⅱ	Tongue	30.7	32.8
9	M	88	ⅣA	Maxillary gingiva	24.4	31
10	F	86	ⅣA	Maxillary gingiva	30.2	34.3
11	M	67	ⅣB	Mandibular gingiva	-	12.7
12	M	73	Ⅲ	Floor of mouth	3.2	20.7
13	M	74	ⅣA	Mandibular gingiva	16.8	20.8
					F: female, M: male	

### Correlation between the number of CTCs and prognosis

**Fig 1:**
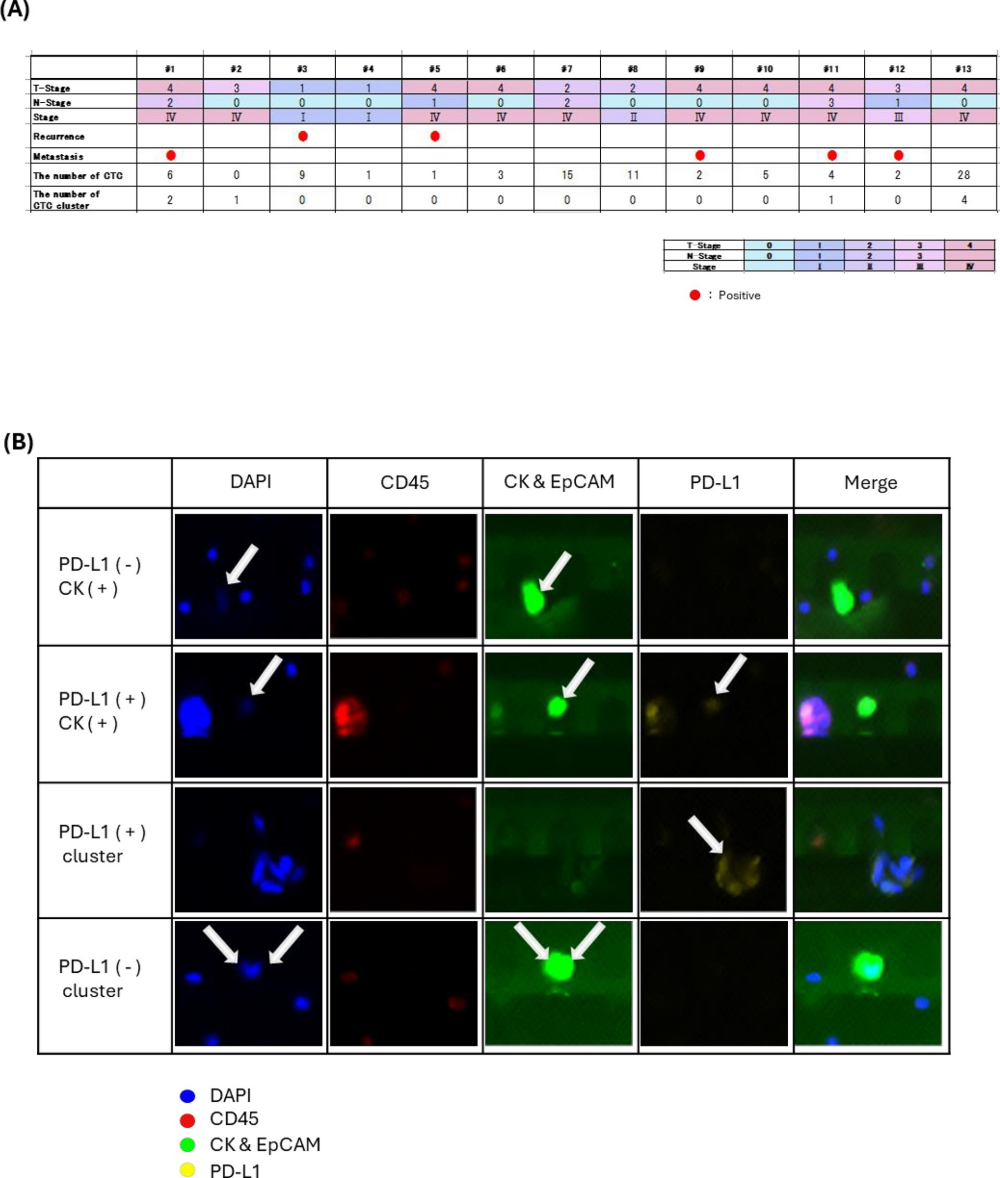
CTC profile and clinicopathological features of 13 patients with OSCC. A: CTC, CTC cluster, profile, and prognosis of each patient. B: Immunohistochemistry images of CTC and CTC clusters. The narrow first line showed PD-L1 negative CTC. The narrow second line showed PD-L1 positive CTC. The narrow third line showed PD-L1 positive CTC clusters. The narrow fourth line showed PD-L1 negative CTC clusters. When we identified that the cells were positive for CK and negative for CD45, they were classified as CTCs or CTC clusters.

**Fig 2:**
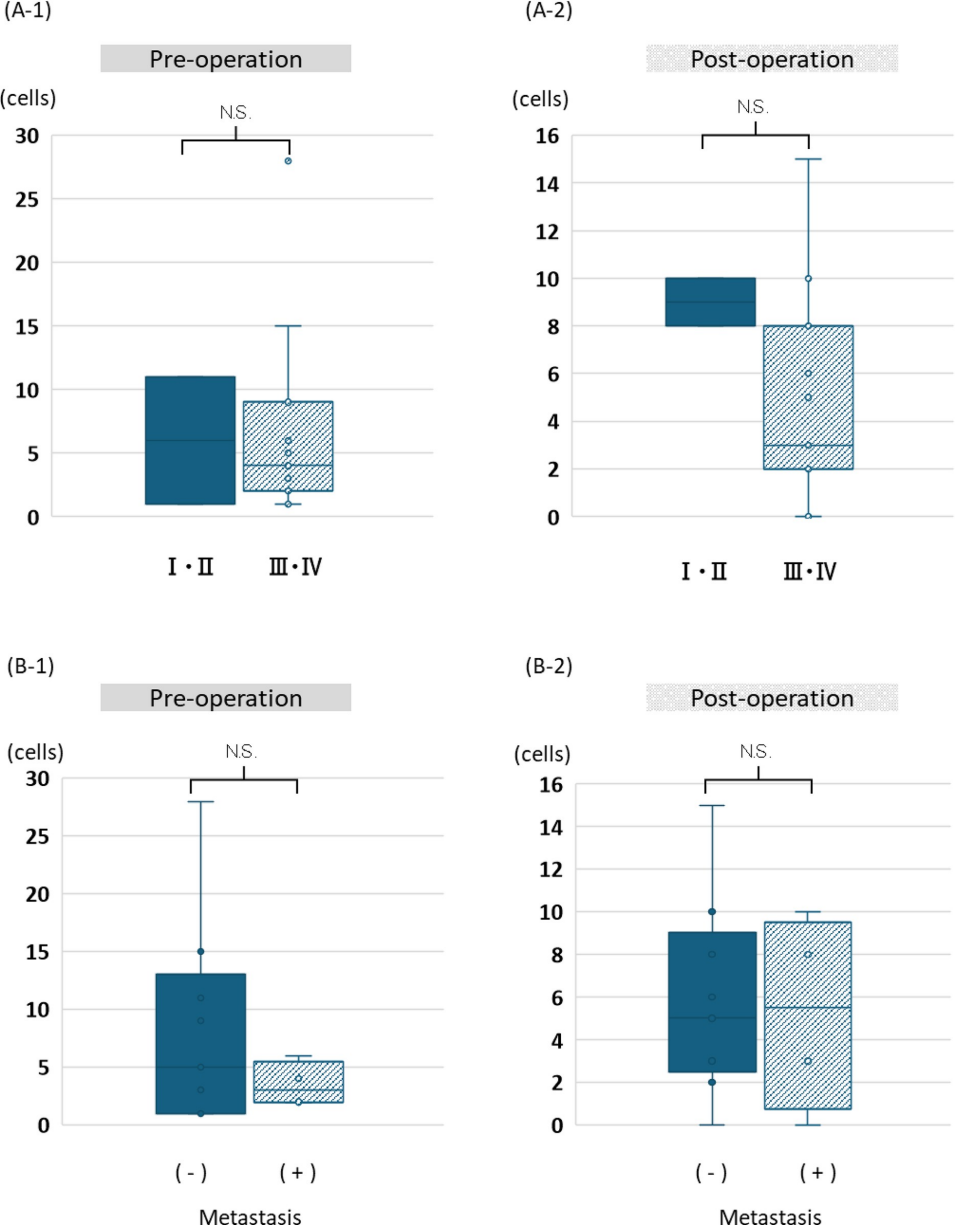
The number of CTC by stage or distant metastasis. A-1 and A-2: No significant difference was observed between the number of CTCs in stage I/II (early) or stage III/IV (advanced) cancer, and in both preoperative and postoperative conditions. B-1 and B-2: No significant differences were observed between the number of CTCs in patients with and without distant metastasis, both preoperatively and postoperatively.

In our study, we detected CTCs in 100% (13/13) of the cases, ranging from 1–32 in number (Fig 1A). Although we stained and separated CTCs (Fig 1B), we did not subdivide them into positive or negative PD-L1 when CTC number analysis was performed, owing to our small sample size. The number of CTCs with distant metastasis was 2–8, and those without distant metastasis were 1–32. The number of CTCs was compared preoperatively and postoperatively, with or without distant metastases and in stage I/II (early) or stage III/IV (advanced) cancer. No significant differences were observed in the number of CTCs between the groups (Fig 2A and 2B). However, we detected CTC clusters in 30.3% (4/13) of the patients, and half of them had distant metastasis (Fig 1A).

### Amount of cfDNA in the advanced-stage group

**Fig 3:**
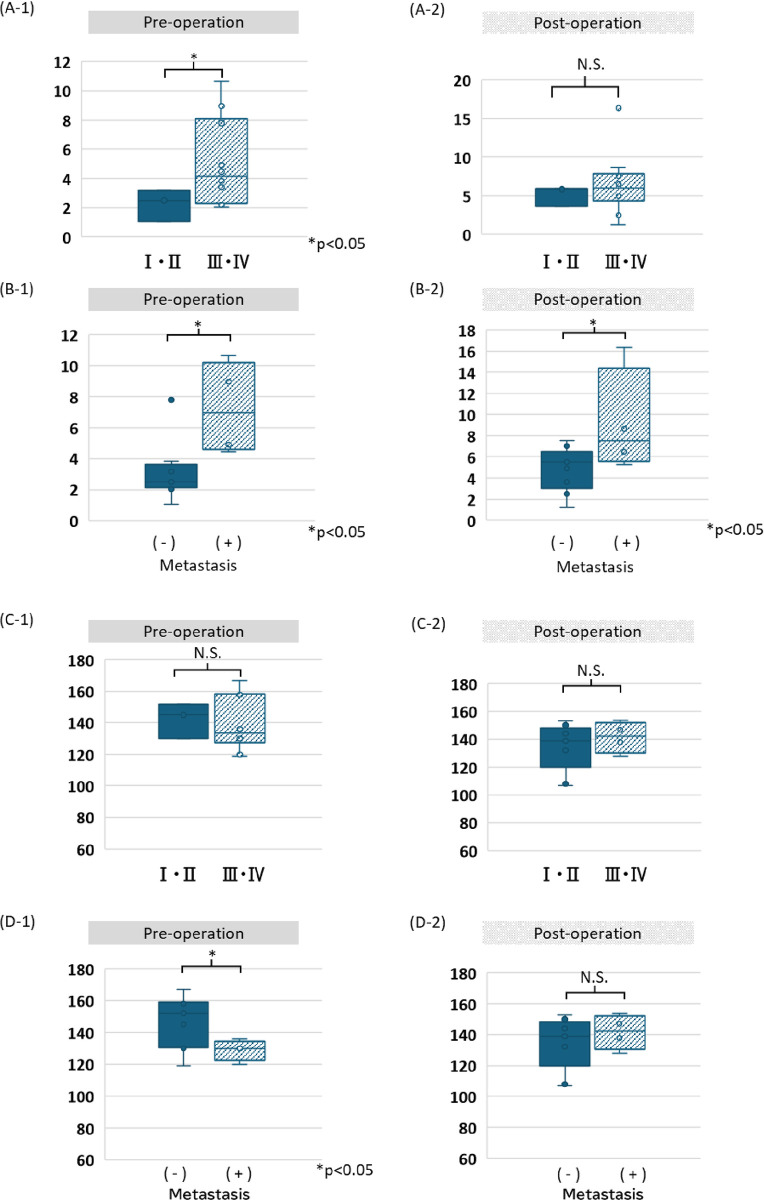
The amount of cfDNA and their fragment size by stage or distant metastasis. A-1 and A-2: The amount of stage III/IV cfDNA is significantly higher than that of stage I/II cfDNA in preoperative conditions, but not significant in postoperative conditions. B-1 and B-2: The amount of cfDNA in patients with distant metastatic cases is significantly higher than that in patients without distant metastasis both preoperatively and postoperatively. C-1 and C-2: No significant differences are observed between the size of cfDNA in stage I/II or stage III/IV cancers, both preoperatively and postoperatively. D-1 and D-2: The size of cfDNA in distant metastatic cases was significantly shorter than that in in non-distant metastatic cases preoperatively, but not postoperatively.

The amount or fragment size of cfDNA was examined in the same manner as the number of CTCs. Preoperatively, the advanced-stage group showed a significantly higher amount of cfDNA than the early-stage group (Fig 3-A1); however this finding was noted postoperatively (Fig 3-A2). The group with distant metastases showed significantly higher amounts of cfDNA than those of the group without distant metastases both preoperatively and postoperatively (Fig 3-B1 and 3-B2).

### Amount of cfDNA and fragment size in the distant metastasis group

No significant differences were observed between the cfDNA fragment size of the early and advanced stages both preoperatively and postoperatively (Fig 3-C1 and 3-C2). Patients with distant metastases preoperatively and postoperatively showed significantly shorter cfDNA fragment sizes than those of patients without distant metastases (p < 0.05, Fig 3-D1); however, this difference was not observed postoperatively (Fig 3-D2).

### Correlation between NLR, PLR, or CRP and the amount of cfDNA

**Fig 4:**
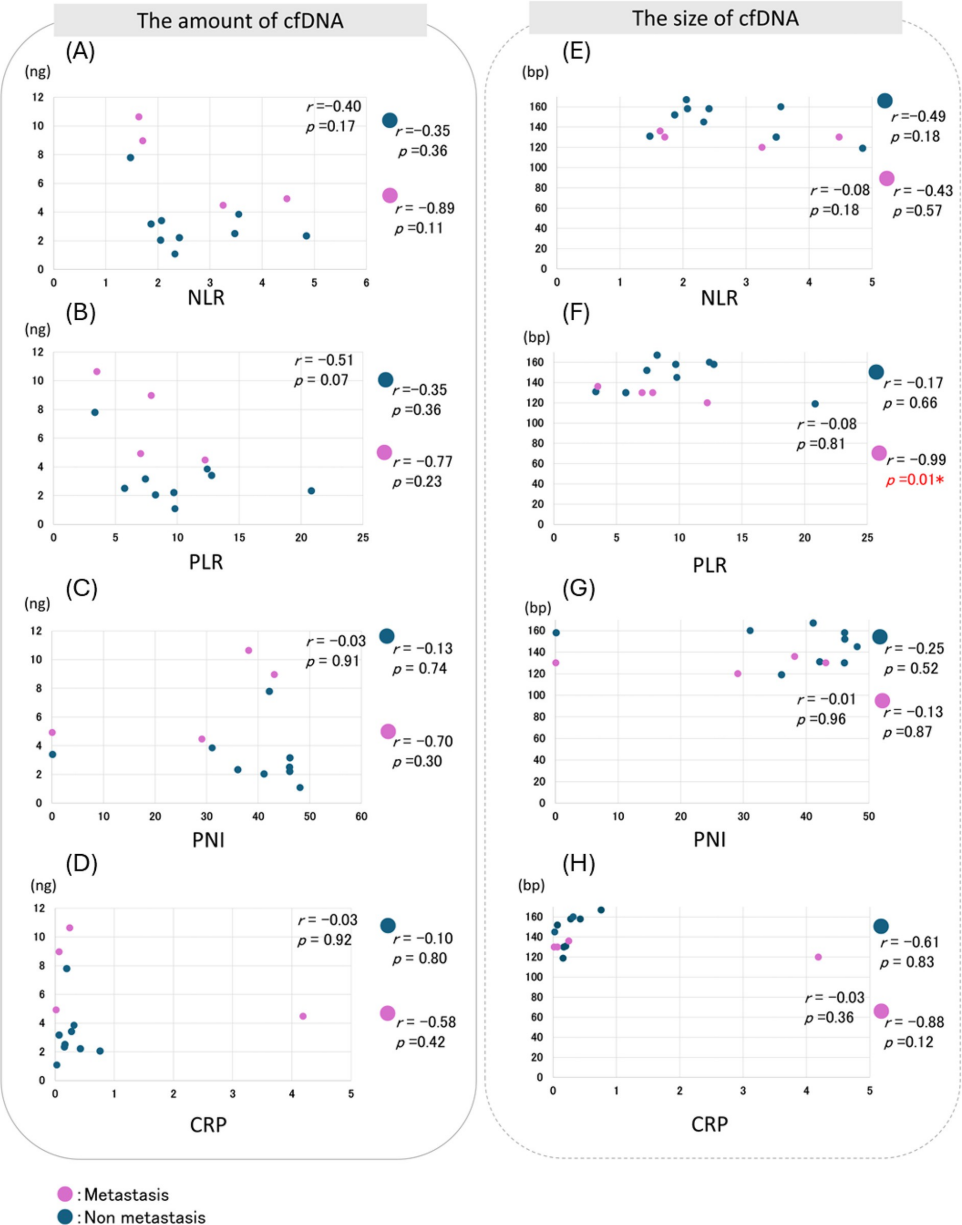
Correlation Analysis of IBPS and cfDNA in target patients. NLR, PLR or CRP and the amount of cfDNA were negatively correlated, but not correlated with the size of cfDNA.

The relationships between the amount or size of cfDNA and NLR, PLR, and PNI were analyzed. Negative correlations were observed between the cfDNA amount of the NLR, PLR, and CRP (Fig 4). However, no significant correlation was observed between the cfDNA fragment size and IBPS.

### Mutations in 15 genes detected in the NGS of cfDNA

**Fig 5:**
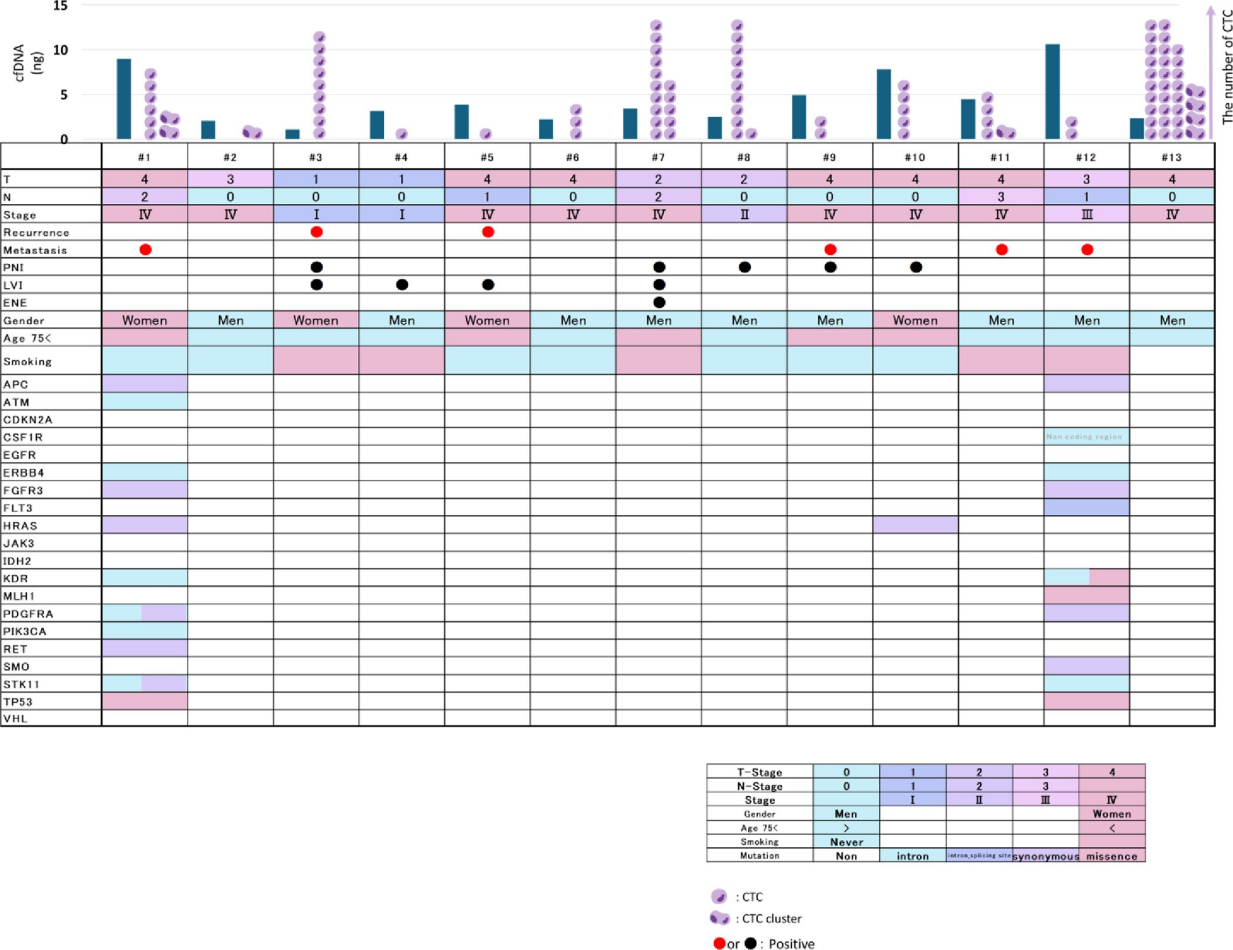
Mutation Landscape and clinicopathological features of 13 patients with OSCC. The clinical details of all recruited patients are shown in the figure. Perineural Invasion (PNI), Lymphovascular Invasion (LVI), Extranodal Extension (ENE).

cfDNA mutations were detected in 15 genes (27 mutations) of three recruited patients (Fig 5). The details are as follows: 15.4% (2/13) for *APC*, *ERBB4*, *FGFR3*, *HRAS*, *KDR*, *PDGFRA*, *STK11*, and *TP53*, and 7.7% (1/13) for *ATM*, *CSF1R*, *FLT3*, *MLH1*, *PIK3CA*, *RET*, and *SMO*. One common mutation in the two patients was *P53_c*.*215C>G_p*.*Pro72Arg (rs1042522)* which was registered as a single nucleotide polymorphism (SNP). In the #8 patient, 11 of 12 mutations were single nucleotide variants (SNVs). The remaining intron variant was *ATM_c*. *8850 +22_8850 +23 insT*. In the #12 patient, only one *HRAS* SNV was detected: *HRAS_c*.*81T>C (p =)*, *rs12628*. In the #15 patient, 12 of 14 mutations were SNV. The remaining two genes were C*SF1R_chr5*:*150054033–150054034 TG>GA (rs386693509)* and *KDR_c*.*798+54G>A (rs3214870)*. The sequencing data generated in the present study may be found in the DDBJ (DNA Data Bank of Japan) BioProject database under accession number (DRA018527.)

## Discussion

In this prospective single-center study, while we did not find any significant associations between the number of CTCs preoperatively and postoperatively and distant metastasis, we did observe an association between the presence of CTC clusters and distant metastases. Additionally, we found that the amount and fragment size of cfDNA were related to distant metastasis.

We detected CTCs in 100% (13/13) of the cases, and the number of CTCs was 1–32. The number of CTCs with distant metastasis was 2–8 and those without distant metastasis were 1–32. In another report, the number of CTCs was 1–43 [10–12, 26–28] and their detection rate was 5.5–80% [10–12, 26–29]. In their report, Inhestern et al. reported that the median number of CTCs was 3295 per mL [29], which is approximately 2000 times higher than that reported in other studies. Results vary widely depending on the antibody or detection device used. Garitaonaindia et. al. also reported that CTC analysis cannot be recommended as a reliable biomarker for minimal residual disease, showing that their CTC data in 180 patients with non-small cell lung cancer did not correlate with overall survival or disease-free survival [30]. No consistent data is available on the number of CTCs and their prognosis; however, expectations for CTC analysis are rising as they may be a source of complete information regarding cancer status. In addition, Lin et al. successfully cultured CTC in vitro [31], a technical improvement that may contribute to the diagnosis and treatment of cancer.

We detected CTC clusters in 30.3% (4/13) of the patients, half of whom had distant metastasis. CTC clusters are not simply a group of tumor cells. Their microenvironment includes immune cells, platelets, dendritic cells, cancer-associated fibroblasts, and tumor stroma [32]. The presence of CTC clusters is associated with poor clinical outcomes in many types of cancers such as breast cancer [32], prostate cancer [32], small cell lung cancer [33], melanoma [34], gastric cancer [35], colorectal cancer [36], liver cancer [37], pancreatic ductal adenocarcinoma [38], and renal cancer [39]. Nasr and Lynch reported that CTC clusters can adapt in tiny capillaries during a metastatic cascade because they are bigger compared with a single CTC, travel slower and closer to the endothelium, allowing for quicker extravasation, and are more resistant to shear stress compared with single CTCs through cellular, molecular differences [40]. Maeshiro et al. showed that CTC clusters are resistant to anoikis under fluid shear stress conditions in an E-cadherin-dependent manner, which leads to distant colonization in head and neck squamous cell carcinoma (HNSCC) cell lines [41].

In this study, the distant metastasis group exhibited a significantly high cfDNA level. In patients with esophageal squamous cell carcinoma, cfDNA levels were significantly higher in patients with distant metastasis than in patients without distant metastasis [42]. In the long term, cfDNA levels reduced in response to therapy and increased during recurrence [19, 43, 44]. These results suggest that cfDNA is a useful tool for monitoring disease conditions.

Additionally, the distant metastasis group showed a significantly short cfDNA fragment size. Underhill et al. suggested that a specific subset of cfDNA fragment lengths may be derived from tumors, which shows that mutation (epidermal growth factor receptor in lung cancer, BRAF in melanoma) allele frequencies increased in shorter fragments of cfDNA [45].

Of the 15 mutations detected in this study, only three genes (*TP53*, *HRAS*, and *MLH1*) were associated with pathogenicity. The *TP53_c*.*215C>G_p*.*Pro72Arg (rs1042522)* mutation was registered as an SNP. Pinheiro et. al. reported that *TP53_c*.*215C>G_p*.*Pro72Arg* has an increased odds ratio in HNSCC; however, it does not relate to its prognosis [46]. However, in our study, two patients (#1 and #15), who commonly possessed *TP53_c*.*215C>G_p*.*Pro72Arg* showed short-term distant metastasis. In addition to oral cancers, many other mutated genes, including HRAS and MLH1, exist in other cancer types. *HRAS_c*.*81T>C(p*. *=) (rs12628)*, detected in #8 patients, has been reported as a pathogenic SNV, [47] in colorectal cancer [48], melanoma [49], and thyroid carcinoma [50]. *MLH1_ c*.*1151T>A_p*.*V384D (rs63750447)*, detected in the #12 patient, is probably a damaging SNV in lung cancer [51] and colorectal cancer [52]. Chiu et al. reported that in *EGFR_L858R* positive lung adenocarcinoma, *MLH1_ p*.*V384D* was associated with a poor response to EGFR tyrosine kinase inhibitors [51]. In distant metastases in two adolescents and young adults with tongue cancer (though we eliminated these cases in this study because that was a preliminary study), we detected common mutations in *TP53* and *CDKN2A* both in the cfDNA derived from the distant metastatic condition and DNA derived from the formalin-fixed paraffin-embedded primary cancer tissue. In this study, we expected to detect mutations in both *TP53* and *CDKN2A* in patients with poor prognosis. In contrast, we detected mutations in 50% (2/4) of *TP53* and 0% (0/4) of *CDKN2A* in the four distant metastatic cases. Currently, we are examining the relationship between immunity and the cancer microenvironment, which will be a topic for future research.

This study had some limitations owing to its small sample size and short-term follow-up. However, reports on the relationship between CTC or cfDNA and distant metastasis in OSCC are not available. Further large-scale studies with long-term follow-ups are required to confirm our findings.

## Conclusions

The increased number of CTC clusters and cfDNA would be reliable factors for the diagnosis of OSCC and clinically significant for future treatment.
